# Recent Progress in Nanoparticle-Based Biosensors for Monitoring *Shigella* spp. in Food Safety: A Critical Review

**DOI:** 10.3390/bios16080435

**Published:** 2026-08-11

**Authors:** Sumeyra Savas, Seyed Mohammad Taghi Gharibzahedi

**Affiliations:** 1 Biosensors and Biotechnology Laboratory (BBL), Medical School, Bandırma Onyedi Eylül University, Bandırma 10200, Balıkesir, Türkiye; 2 Institute of Materials Science, Faculty of Engineering, Kiel University, 24143 Kiel, Germany; smg@tf.uni-kiel.de

**Keywords:** *Shigella* species, foodborne pathogen detection, nanobiosensors, gene-level biosensing, point-of-care detection, food contamination monitoring

## Abstract

*Shigella* is a foodborne bacterial pathogen with a low infectious dose and significant public health impact. Culture-based and molecular techniques provide reliable identification but are time-consuming. Nanoparticle-based biosensors offer sensitive, selective, and compact alternatives. Recent advances in nanoparticle-based biosensors for *Shigella* spp. (*S. flexneri*, *S. sonnei*, *S. dysenteriae*, and *S. boydii*) detection have been reviewed in terms of signal amplification, biorecognition, biological targets, sensor types, and performance in real food matrices. Detection strategies rely on gene-level and whole-cell recognition. Targeting virulence genes, invasion plasmid antigen H (*ipaH*), provides stable genus-level identification, whereas whole-cell recognition facilitates rapid detection without extensive sample preparation. Optical biosensors, including fluorescence-based methods, surface-enhanced Raman spectroscopy (SERS), and localized surface plasmon resonance (LSPR), achieve low detection limits with strong tolerance to complex food matrices. Electrochemical biosensors offer operational simplicity, portability, and suitability for food screening. Lateral flow and hybrid systems provide rapid detection through simplified assay formats and visual readout, with performance influenced by the balance between speed and sensitivity. Validation in real food matrices shows acceptable recoveries, minimal cross-reactivity, and agreement with reference methods. This overview provides a design-oriented framework for nanoparticle-based biosensor selection in food safety by integrating nanomaterial function, biosensor design, and performance characteristics.

## 1. Introduction

Foodborne illnesses represent a major global public health concern and lead to considerable morbidity, mortality, and economic losses in both developed and developing nations [1,2]. The food supply chain faces ongoing contamination challenges as a result of expanding global food production, complex distribution networks, and insufficient hygiene control at certain processing and handling stages [3,4]. A broad range of food matrices has been associated with foodborne outbreaks, including fresh fruits and vegetables, meat and dairy products, drinking water, and ready-to-eat foods that receive minimal processing and are often consumed without additional heat treatment [5,6]. These food categories are more prone to large-scale outbreaks because they are more vulnerable to microbial contamination during harvesting, processing, storage, and handling after processing [7,8]. The burden of foodborne infections has increased globally in recent years due to the rapid emergence and spread of antibiotic-resistant bacteria, which has also decreased the efficacy of traditional treatments [9,10]. The growing prevalence of antibiotic resistance has led to greater demand for improved food monitoring and surveillance systems aimed at timely detection, precise identification, and risk control of foodborne pathogens (FBPs) [11].

*Shigella* spp. are Gram-negative, facultative intracellular enteric bacteria and rank among the main bacterial causes of bacillary dysentery worldwide. The genus includes four species, *S. dysenteriae*, *S. flexneri*, *S. boydii*, and *S. sonnei*, which together account for a large share of diarrheal disease, mainly in low- and middle-income countries with limited water, sanitation, and hygiene infrastructure [12,13]. The fecal-oral route involves contaminated food or drink as well as direct person-to-person contact. This is the main infection method in areas with high population density [14]. Consuming extremely few bacterial cells (usually less than 10–100) can lead to *Shigella* infections, increasing the risk of an epidemic and complicating prevention and control procedures. The global spread of antimicrobial-resistant *Shigella* strains has raised the illness burden and reduced treatment choices, underscoring the necessity for highly sensitive and early detection methods in food safety monitoring and inspection [12,15].

Decisions are delayed during surveillance and epidemic response operations because enrichment, isolation, and biochemical confirmation methods used in conventional diagnosis involve multiple steps and require significant time and effort [16]. The regulatory standard for confirming the presence of *Shigella* in food products is still based on culture-based procedures. Rapid screening in food service and catering settings is essential to control cross-contamination risks and prevent further transmission, but long processing times create a significant operational burden [17]. Although molecular techniques, such as polymerase chain reaction (PCR) and quantitative PCR (qPCR), improve analytical sensitivity and shorten turnaround times, their application outside laboratory settings is restricted because they require specialized equipment, skilled personnel, and controlled laboratory conditions [1,14]. In complex food matrices, inhibitors and coexisting matrix components can interfere with amplification-based signal outputs when adequate controls and careful sample preparation are lacking [18,19]. Immunological assays such as enzyme-linked immunosorbent assay (ELISA) permit faster analysis, although high cross-reactivity and limited sensitivity at low target levels remain concerns, particularly when closely related enteric bacteria share similar antigenic traits [20]. Another problem is viable-but-non-culturable (VBNC) cells, which are difficult to identify using growth-dependent techniques and make risk assessment more difficult when confirmation depends only on culturability [21,22,23]. These drawbacks emphasize the necessity of quick, precise, and food-safe *Shigella* detection techniques.

Biosensors are analytical tools that combine biological recognition components, like aptamers, nucleic acids, or antibodies, with transducers that translate recognition events into quantifiable signals. Biosensors have useful advantages for food hazard monitoring as compared to culture-based and molecular methods, such as shorter analysis times, ease of use, and compatibility with small device topologies [24,25,26,27,28]. Nanoparticles (NPs) are key elements in biosensors to improve signal transduction and analytical sensitivity. Their high surface-to-volume ratio allows for dense immobilization of biodetection elements, leading to stronger interaction with the target and enhanced signal output [29,30]. The optical, electrical, and magnetic properties of NPs accelerate efficient electron transport and photoresponse, both of which are essential for sensitive pathogen measurement. These material properties make NP-based biosensors appropriate for *Shigella* detection in food safety surveillance since they are linked to reduced detection limits (LODs), quicker reaction times, and increased resistance to matrix-related effects [31,32,33,34].

**Figure 1:**
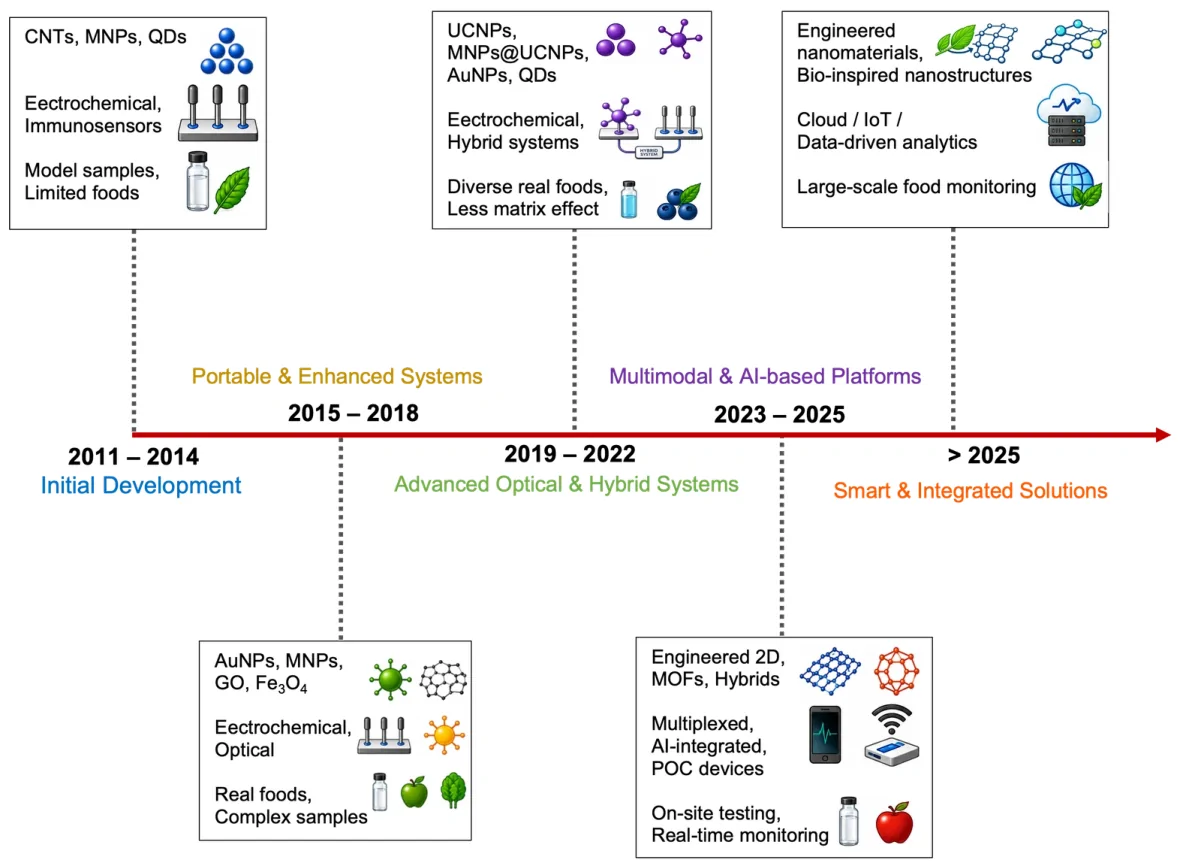
Roadmap of technological progress in NP-based biosensors for *Shigella* detection.

The literature has described a number of NP-based biosensors for *Shigella* spp. that are directly related to food monitoring. Designs that have been reported deal with species-level identification of *S. flexneri*, *S. sonnei*, and *S. dysenteriae* as well as virulence-marker detection, such as invasion plasmid antigen H (*ipaH*), in technology-focused studies. Published work also covers multiplex detection for simultaneous screening of foodborne bacterial pathogens, alongside electrochemical aptasensors, optical and colorimetric assays, and fluorescence-based designs. The breadth of these reported designs reflects methodological diversity in food-testing practices [29,31,35,36,37,38,39]. The technological progression of NP-based biosensors for *Shigella* detection is summarized in Figure 1, presenting major advances in nanomaterials, biosensor platforms, and applications in food matrices.

Although biosensor research is growing rapidly, most available review publications either discuss NP-based biosensors from a general technological viewpoint or consider FBPs in a broad scope, with limited focus on individual pathogens or targeted food monitoring applications [25,40]. Previous publications have mainly summarized nanomaterial-enabled biosensors from a general technological perspective or discussed *Shigella* along with other FBPs. In contrast, the current overview specifically focuses on NP-based biosensors developed for *Shigella* detection in food safety and evaluates the relationship among nanomaterial function, biosensor design, biological target selection, analytical performance, and practical applicability in a unified design-oriented framework. Publications addressing *Shigella* spp. as a primary subject are scarce, whereas those including *Shigella* present it as one component of broader pathogen panels without providing an in-depth, pathogen-specific analysis for food safety applications. Published evidence regarding NP-assisted biosensors for *Shigella* detection in food matrices, practical monitoring requirements, and validation using real samples is currently insufficient for comprehensive comparison of nanomaterial selection, biorecognition choices, LODs, and suitability for complex food environments. A focused assessment of recent work on NP-based biosensors for *Shigella* spp. detection in food control is therefore warranted. The present review summarizes recent developments in NP-based biosensors reported for *Shigella* spp. detection in food quality and safety assessment. The types of nanomaterials, sensor designs, signal transduction modalities, and stated detection results from actual food samples are all studied. Accompanied by research directions pertinent to routine food monitoring and safety control, current technical limitations and unresolved concerns are also explained.

**Figure 2:**
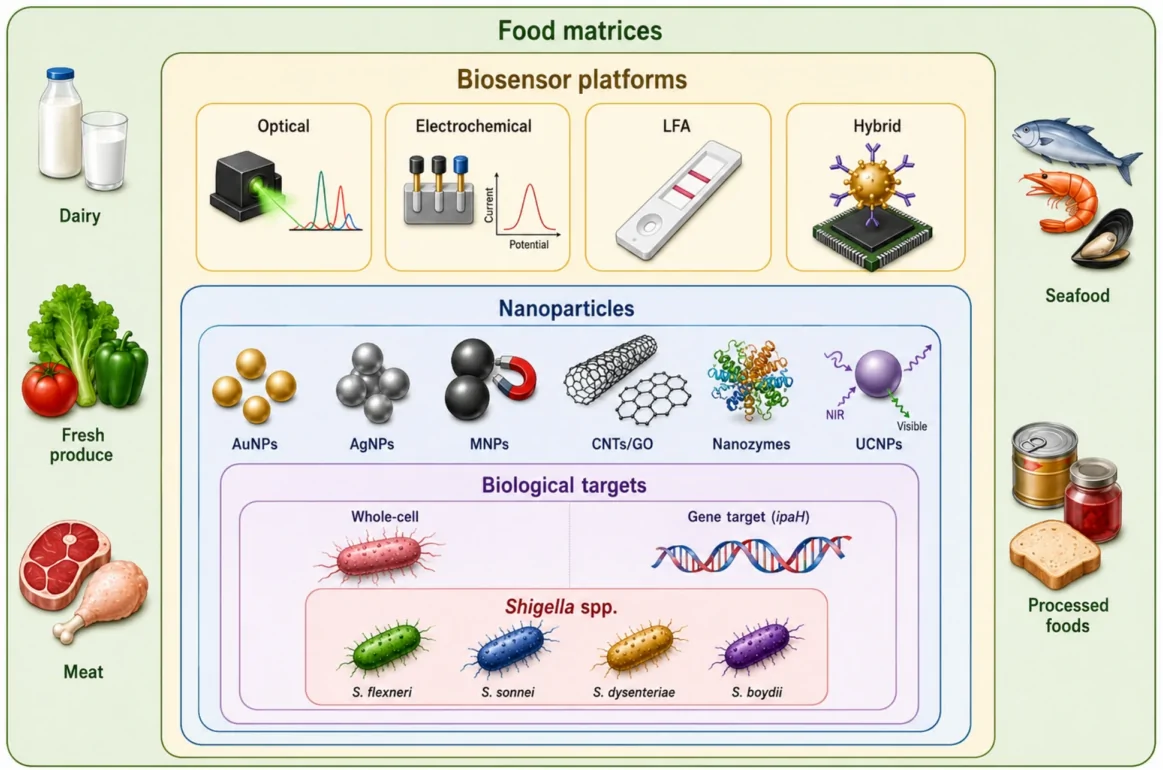
Schematic overview of the design-oriented framework discussed in this review, illustrating the relationship among *Shigella* biological targets, NP classes, biosensor platforms, and representative food matrices for food safety monitoring.

This review moves beyond conventional classifications based solely on sensing modality or signal type and focuses on the relationship between *Shigella*-specific biological characteristics and biosensor design strategies. It examines target selection (whole-cell versus gene-level detection), matrix-dependent performance, and application-oriented sensor configuration. By linking nanomaterial function to pathogen-specific detection requirements, this work defines a design-oriented framework that establishes the relationship between biosensor performance and the unique properties of *Shigella*, instead of approaching it as a generic FBP. This perspective clarifies inconsistencies among reported studies and guides the selection of sensing strategies for food safety applications. Understanding the biological characteristics of *Shigella* provides the basis for interpreting the design principles of NP-based biosensors. Figure 2 depicts the conceptual relationships among *Shigella* biological targets, NP classes, biosensor platforms, and representative food matrices discussed throughout the following sections.

## 2. *Shigella* spp. in Food Safety-Oriented Biosensing

From a biosensor development perspective, *Shigella* species have biological and molecular characteristics that influence sensor configuration, target selection, and detection outcomes. *Shigella*, unlike many FBPs, is restricted to human hosts and has no known animal or environmental reservoirs. This host specificity emphasizes contamination events associated with food handling and processing activities involving human contact [12,14]. As a result, biosensor design has focused on molecular markers shared among *Shigella* spp. that are absent in symbiotic or closely related enteric bacteria. The molecular characteristics of *Shigella*, including its conserved virulence-associated genetic elements and surface antigens, provide selective biological targets for NP-based biosensors using antibodies, aptamers, or nucleic acid probes. Both genus-level and species-specific targets have been investigated for different analytical purposes in food safety applications [12,14,18].

Additionally, experimental evidence suggests that *Shigella* can endure physiological stress circumstances that make standard analytical tests more difficult. Under such circumstances, binding interactions and signal production are impacted by decreased metabolic activity and altered surface characteristics [21]. These results show the importance of biosensing techniques that can identify functional biomarkers, intact cells, or nucleic acids without depending on culturability [18,41].

## 3. Functional Roles and Transduction Mechanisms of Nanomaterials in *Shigella* Detection

In this review, nanomaterials are discussed according to their primary functional contribution within NP-based biosensors. Because a single nanomaterial may simultaneously participate in target capture, probe immobilization, signal generation, and signal amplification, these categories are intended to represent the principal function emphasized in the corresponding biosensing platform, not mutually exclusive classifications. The optical and electrochemical subsections present the transduction mechanisms underlying signal generation in these NP-based biosensing strategies.

### 3.1. Nanomaterial-Mediated Signal Enhancement

Numerous NP-assisted designs that convert low target abundance into detectable responses indicate that signal amplification is a key function of nanomaterials in *Shigella* biosensing. Gold nanoparticles (AuNPs) contribute to signal generation through plasmonic effects [32,42]. DNAzyme-driven colorimetric systems further translate target recognition into catalytic cleavage events, producing measurable optical responses [39]. Fluorescence- and surface-enhanced Raman spectroscopy (SERS)-based strategies similarly rely on nanomaterial-dependent signal generation [34,43], including energy transfer processes [34] and electromagnetic hot-spot formation on NP surfaces [43].

Plasmonic AuNPs amplify sensing signals with localized surface plasmon resonance (LSPR) [42,44]. Fluorescent nanomaterials, including upconversion nanoparticles (UCNPs), operate through anti-Stokes emission, where near-infrared excitation is converted into visible emission, thereby reducing background autofluorescence in complex matrices [34,45]. In electrochemical systems, nanomaterials such as AuNPs and carbon-based nanostructures facilitate electron transfer by increasing effective electrode surface area and conductivity, which supports higher probe density and measurable changes in interfacial charge transfer [24,35,46,47,48,49]. In general, the analytical advantages of these nanomaterials arise from their low-dimensional characteristics, including high surface-to-volume ratio, quantum confinement effects, and distinct electronic and optical properties, which enhance bioreceptor immobilization, signal amplification, and sensitive transduction [50,51]. Nevertheless, biosensor performance is determined by the composition, morphology, and surface functionalization of nanostructures [52]. Precise control of the structure-property relationship is difficult, whereas inappropriate immobilization may cause uncontrolled anchoring of biomolecules and compromise target recognition [51]. Moreover, nonspecific components in complex samples, potential toxicity, biodegradation, and challenges in large-scale fabrication continue to limit practical applications [50,52].

Besides analytical sensitivity, batch-to-batch reproducibility is a critical factor governing the practical reliability of NP-based biosensors. Variations in nanomaterial synthesis, surface functionalization, electrode modification, and biorecognition elements may affect signal output, analytical precision, and sensor-to-sensor consistency, limiting standardization and large-scale implementation [53,54,55]. Reproducible fabrication protocols, controlled surface chemistry, rigorous quality assessment, and consistent immobilization of recognition elements are required to achieve comparable analytical performance across production batches and laboratories [56,57,58,59]. Practical performance is also influenced by food-specific conditions, including matrix composition, biofouling, pH variation, and matrix compatibility, which may alter biosensor stability and analytical reliability when real food samples are analyzed [60,61].

**Figure 3:**
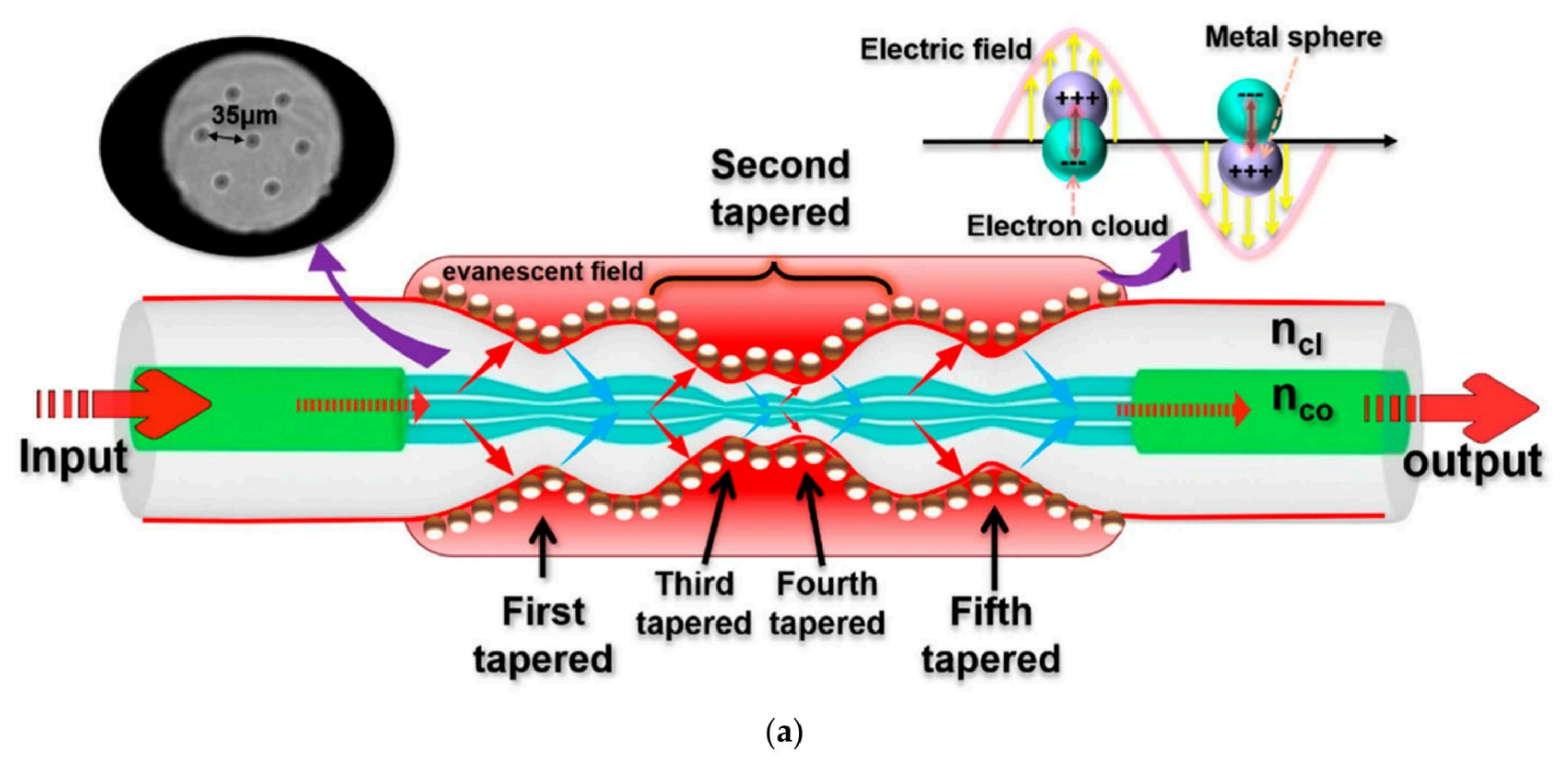

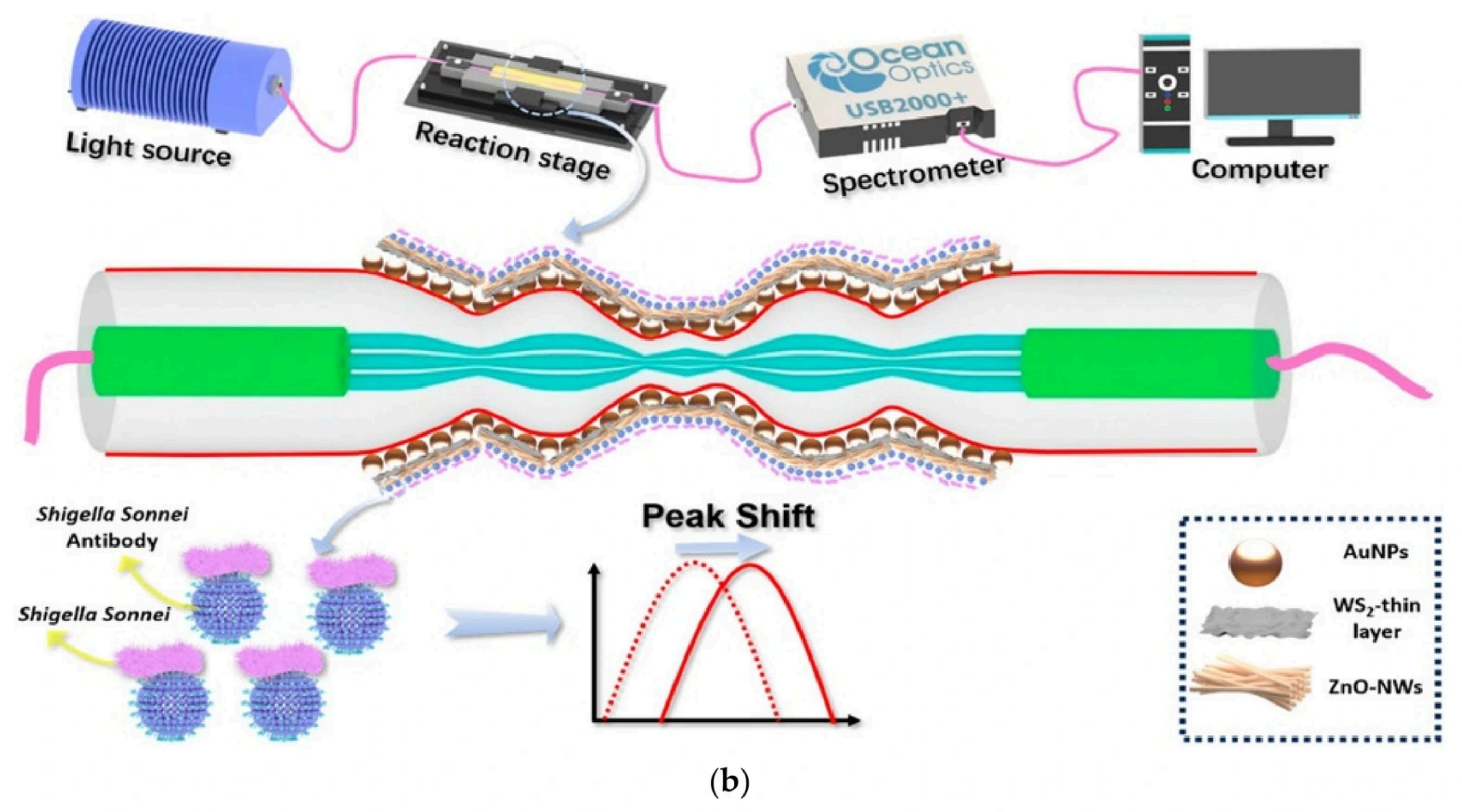
Schematic representation of (**a**) the working principle of an LSPR-based crayfish-type optical fiber biophotonic sensor and (**b**) the experimental setup for detecting *S. sonnei* using a multimodal LSPR-enhanced crayfish-type optical fiber sensor based on hybrid nanomaterials. Reproduced from Zhang et al. [48] under the terms of the Creative Commons Attribution (CC BY) license.

Figure 3a,b illustrate the operating principle of an LSPR-based optical fiber biosensor for *Shigella* detection. In addition, sample conditions should be considered when evaluating analytical performance because food samples vary considerably in their physicochemical properties and microbial composition. Variations in bacterial concentration, physiological state, sample preparation, and pre-enrichment procedures may affect probe accessibility, signal generation, and assay reproducibility. Injured and VBNC *Shigella* cells, along with indigenous microbiota, can reduce recovery efficiency and analytical accuracy during food analysis. Appropriate sample preparation and validation in representative food matrices are necessary to ensure reliable biosensor performance under practical conditions [62].

### 3.2. Surface Functionalization and Biorecognition Enhancement

Surface functionalization of nanomaterials has been widely applied to improve biorecognition specificity and stability in *Shigella* biosensing platforms. AuNP-modified electrodes have been used as immobilization matrices for probe attachment and signal enhancement in *Shigella* biosensing platforms, supporting stable biorecognition interfaces [63]. In these systems, AuNP functionalization facilitated effective probe–target hybridization and improved electrochemical or optical signal responses [35,63]. Hybrid nanomaterial platforms incorporating magnetic nanoparticles (MNPs) and AuNPs have also been functionalized with nucleic acid probes or fluorescent labels to enhance selective recognition of *Shigella* spp. Elahi et al. [64] demonstrated that probe immobilization on iron oxide (Fe_3_O_4_) and AuNP-based substrates supported stable fluorescence-based detection with improved specificity. The sensing strategy involves stepwise surface functionalization of MNPs and AuNPs with DNA probes, followed by target-induced sandwich complex formation, magnetic separation to remove nonspecific species, and subsequent release of fluorescently labeled probes, resulting in amplified and selective fluorescence signal output.

Aptamer-functionalized nanostructures have also strengthened target selectivity, as reported in fluorescence- and SERS-based sensing platforms for *S. flexneri*, where optimized aptamers were immobilized on NP surfaces to achieve specific binding [65,66]. He et al. [66] prepared fluorescent probes and SERS tags via sequential NP functionalization with signal reporters and *S. flexneri*-specific aptamers. Upon target recognition, NP–bacteria assemblies were formed, enabling metal-ion-assisted signal amplification and dual fluorescence/SERS readout for sensitive detection. In addition, genosensing strategies employing AuNP-modified substrates have mediated direct recognition of *Shigella* genetic markers, including the *Shigella* plasmid antigen (*spa*) gene, by facilitating probe attachment and enhancing hybridization efficiency [63]. Generally, NP surface functionalization plays a central role in stabilizing biorecognition elements and improving selective target recognition in *Shigella* biosensing applications. It should be noted that selective identification of *Shigella* spp. originates from the specificity of the biorecognition element, not from the transduction mechanism. Antibodies, aptamers, and nucleic acid probes directed toward conserved virulence genes (e.g., *ipaH*) or species-specific surface structures are responsible for target recognition. In contrast, nanomaterials contribute to optical, electrochemical, fluorescent, or magnetic signal generation after the recognition event has occurred. Accordingly, the transducer increases analytical sensitivity, whereas discrimination between *Shigella* spp. and non-target microorganisms depends on the molecular recognition process.

### 3.3. Optical Signal Enhancement Mechanisms

Optical signal enhancement in *Shigella* biosensing is achieved by plasmonic, fluorescence, and Raman-based sensing approaches [42,67,68]. Aggregation-induced plasmon coupling in AuNP systems produces measurable optical shifts, forming the basis of colorimetric detection strategies in *Shigella* biosensing [42]. Plasmonic responses can also be modulated by environmental conditions such as pH, leading to rapid optical detection through signal variation [36].

**Figure 4:**
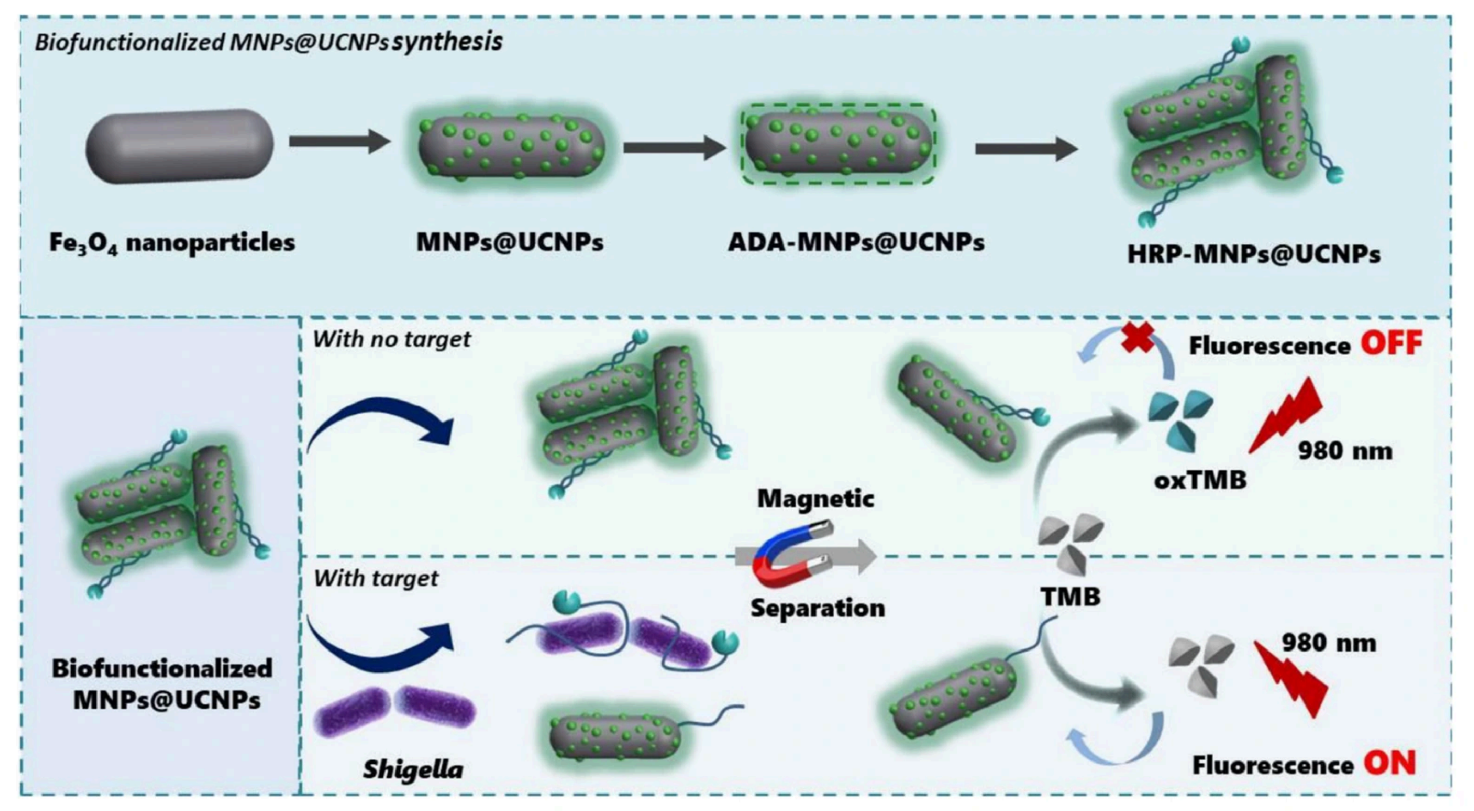
Schematic illustration of the fabrication of the biofunctionalized MNPs@UCNPs nanoplatform and its optical signal enhancement mechanism for *Shigella* detection (reproduced from Song et al. [46]).

A hybrid nanostructure based on biofunctionalized MNPs@UCNPs has been developed for *Shigella* detection, using magnetic enrichment before fluorescence-based detection to improve sensitivity and reduce matrix interference [46]. Figure 4 illustrates the fabrication of the biofunctionalized MNPs@UCNPs platform and its optical signal enhancement mechanism for *Shigella* detection. SERS has also been applied as a powerful optical enhancement approach, where AuNP dimers or silver nanoparticle (AgNP) substrates generate electromagnetic hotspots for ultra-sensitive detection. The schematic illustrates the stepwise dilution of *Shigella* cultures to low bacterial concentrations and their subsequent detection using AgNP-based SERS substrates. AgNPs deposited on filter paper form a highly adhesive plasmonic surface, where laser excitation generates enhanced Raman signals from bacteria trapped between AgNPs, enabling rapid and sensitive detection down to low colony-forming unit (CFU) levels [69].

Recent studies have demonstrated that LSPR-enhanced optical fiber platforms based on hybrid nanomaterials, including AuNPs combined with molybdenum disulfide (MoS_2_) or copper sulfide (CuS)-based nanocomposites, enable label-free and real-time detection of *Shigella* species, supporting their applicability within food-testing workflows [48,67,68].

### 3.4. Electrochemical Enhancement Mechanisms

Electrochemical enhancement strategies for *Shigella* biosensing rely on nanomaterial-modified electrodes to improve probe immobilization, charge-transfer efficiency, and measurable electrical responses following target binding. The step-by-step development of an electrochemical DNA biosensor for *S. flexneri* detection in food samples was conducted, including DNA extraction, electrode surface modification, target DNA hybridization, and electrochemical signal detection. DPV-based sensing utilized AQMS (anthraquinone-2-sulfonic acid monohydrate sodium salt) as a redox indicator, where changes in peak current before and after hybridization with complementary *S. flexneri* DNA provide sensitive and quantitative analysis [24]. Electrochemical detection strategies based on AuNP-modified electrodes monitor changes in charge-transfer resistance or impedance following target binding, facilitating sensitive and selective detection of *Shigella* species in complex sample matrices [35]. Ag-decorated nanobeads integrated into a flexible aptasensor generated enhanced electrochemical signals for *S. dysenteriae*, contributing to low-cost and portable detection concepts relevant to food-testing situations. Paper-based electrochemical systems have also received attention in this area [38].

Carbon-based nanomaterials have been incorporated into electrochemical biosensors to improve electron transfer and signal generation. For example, a disposable immunosensor using a multiwalled carbon nanotube–sodium alginate (MWCNT/SA) composite electrode was developed for *S. flexneri*, providing rapid electrochemical responses suitable for screening applications [70]. A hybrid electrochemical biosensing platform combining porous biochar, ultrathin MoS_2_ nanosheets, and AuNPs was developed for PCR-free genosensing of *S. dysenteriae*, illustrating the contribution of conductive nanomaterials to direct nucleic-acid detection without amplification steps [71]. Beyond single-target systems, AuNP-assisted multimodal platforms integrating electrochemical readouts have been applied to food-related matrices. The strategy involves thermal DNA extraction from food samples, sandwich hybridization between capture probe-modified MNPs and noble metal NP–labeled reporter probes, followed by magnetic separation and dual electrochemical/inductively coupled plasma–mass spectrometry (ICP-MS) signal readout. An NP-assisted electrochemical and ICP-MS strategy demonstrated sensitive detection of *Shigella* DNA in complex matrices, including food and water samples, underscoring the applicability of electrochemical nanobiosensors to realistic food safety assessment scenarios [49].

### 3.5. Magnetic Target Enrichment and Separation

MNPs mediate target enrichment via external magnetic field-driven separation, increasing local analyte concentration and reducing matrix interference in complex sample matrices [46,47]. Biofunctionalized Fe_3_O_4_ MNPs have been combined with fluorescent nanomaterials to form hybrid platforms (e.g., MNPs@UCNPs; Figure 3), enabling rapid magnetic separation of *Shigella* cells followed by fluorescence-based detection, which improves sensitivity and reduces matrix interference in samples relevant to food and environmental monitoring [46]. A selective reaction to *Shigella* spp. was confirmed by fluorescence nanobiosensors constructed on Fe_3_O_4_ MNPs and AuNPs. Target cell discrimination in liquid samples, indicative of actual testing settings, was made possible by the combination of magnetic manipulation and stable probe attachment [64].

Shorter analysis times in liquid samples were achieved for *S. flexneri* by immunomagnetic configurations based on antibody-coated magnetic particles for selective bacterial capture and concentration before signal transduction [72]. Using magnetic relaxation measures, MNP-based techniques have also been used to evaluate the metabolic activity of bacteria, including *Shigella* strains. Accordingly, MNP-based biosensors behaved well in optically complex media like blood or nutrient-rich solutions, which were similar to the circumstances found in challenging food and biological samples [47]. These results suggest that MNP-assisted assay designs can improve assay stability, selectivity, and analytical performance in complex food matrices. These functional roles are reflected in the design of NP-based biosensors reported for *Shigella* detection in food-related applications.

The analytical value of nanomaterials cannot be explained by a single physicochemical characteristic. Their role in biosensing systems is characterized by the analytical function assigned to each material, including greater surface availability for bioreceptor immobilization [73,74], optical, electrical, magnetic, and catalytic properties used in signal generation and target recognition [75,76,77,78], as well as target capture, enrichment, and multifunctional sensing configurations [79,80]. Therefore, analytical performance varies with the compatibility between nanomaterial characteristics and the requirements of the biosensing system. Different classes of nanomaterials address different analytical requirements according to the sensing strategy, target type, and sample matrix, indicating that nanomaterial selection should follow the analytical objective and intended application instead of a fixed preference for a single material class [2,3,16].

## 4. Nanoparticle-Based Biosensors for *Shigella* Detection

### 4.1. Optical Nanoparticle-Based Biosensors

#### 4.1.1. Colorimetric Biosensors

Optical NP-based biosensors have been applied for *Shigella* detection using colorimetric formats with visual or spectrophotometric readout. An AuNP-based colorimetric aptasensor detects *S. flexneri* through target-induced NP aggregation, producing visible color changes and a quantitative UV–Vis response (A_700_/A_520_). The assay exhibited a linear detection range of 10^2^–10^6^ CFU mL^−1^ with an LOD of 80 CFU mL^−1^ and a total analysis time of less than 20 min. Validation in smoked salmon samples showed good agreement with plate counting, recoveries of 88.5–110.2%, minimal matrix interference, and detection within 20 min without preconcentration or DNA extraction [31].

A pH-responsive AuNP-based assay detects FBPs through aggregation-induced optical changes under acidic conditions. Acid-induced positive charging of bacterial cells triggered AuNP aggregation and produced distinct plasmonic color changes [36]. For *S. flexneri*, an LOD of 3.3 × 10^2^ CFU mL^−1^ was obtained, corresponding to an optical density of OD_600_ = 0.05. Aggregation-driven color transitions from red to purple or blue could be observed by the unaided eye within 5 min of incubation. The platform was evaluated together with other common FBPs, demonstrating concentration-dependent color responses that enabled semi-quantitative discrimination across different bacterial loads. Although validated in liquid model systems rather than specific food matrices, the simplicity, rapid response time, and instrumentation-free operation demonstrate its potential utility for preliminary screening of pathogenic *Shigella* in food- and water-related samples [36]. Optical genosensing strategies have also been reported at the nucleic acid level. An AuNP-based optical genosensor targets the *spa* gene and detects *Shigella* species through aggregation- or stabilization-dependent optical responses [63]. The platform achieved an LOD of 8.14 ng mL^−1^ and a limit of quantification (LOQ) of 26.6 ng mL^−1^, with a concentration-dependent optical response observed within a linear range of 1.5–80 ng mL^−1^. High sequence specificity was demonstrated using genomic DNA from *S. dysenteriae*, *S. flexneri*, *S. boydii*, and *S. sonnei* as positive targets, while *Pseudomonas aeruginosa*, *Staphylococcus aureus*, *Escherichia coli*, and *V. cholerae* served as negative controls. No significant change in absorbance or color stabilization was observed for non-*Shigella* strains, whereas the presence of complementary *Shigella* DNA maintained the characteristic red color of dispersed AuNPs, confirming high sequence selectivity. Although validated in aqueous model systems instead of real food matrices, the assay’s rapid, instrumentation-free readout was proposed as a cost-effective approach with potential applicability to preliminary food-related screening workflows [63].

#### 4.1.2. Fluorescence and Raman Biosensors

**Figure 5:**
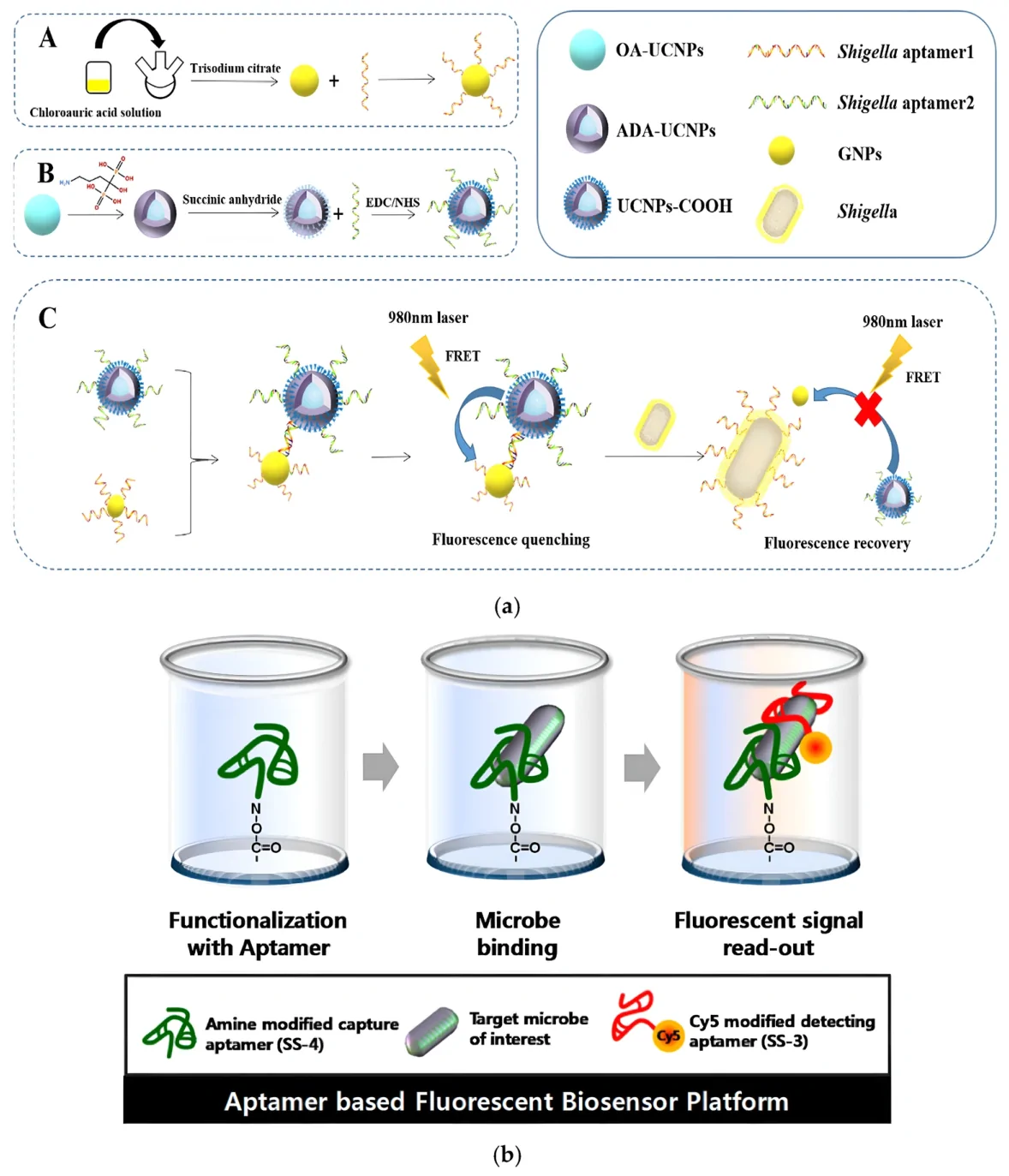
(**a**) Schematic illustration of the fabrication and fluorescence sensing mechanism of the AuNP (GNPs)-UCNP nanoprobe for *Shigella* detection. A–C represent the preparation of the AuNP-based probe, functionalization of UCNPs with the corresponding aptamer, and the FRET-based fluorescence sensing mechanism involving fluorescence quenching and recovery upon target recognition, respectively (reproduced from Chen et al. [34]). (**b**) Principle of *S. sonnei* detection using a sandwich-type aptamer-based fluorescent biosensor, showing sequential capture-aptamer functionalization, target-microbe binding, and fluorescent signal readout using a cyanine 5 (Cy5)-labeled detection aptamer (SS-3) (reproduced from Song et al. [28]). Symbols and colors identifying the sensing components are defined in the respective figure legends.

Fluorescence-based NP biosensors have been developed for sensitive optical detection of *Shigella* in complex food matrices. The fabrication of a UCNP/AuNP-based FRET nanoprobe and its fluorescence sensing mechanism are illustrated in Figure 5a. A UCNP-based Förster resonance energy transfer (FRET) nanoprobe composed of complementary strand-modified UCNPs and aptamer-functionalized AuNPs detects *Shigella* through fluorescence recovery following target binding, with emission at 545 nm under 980 nm excitation [34]. The sensor showed a wide linear range of 1.2 × 10^2^–1.2 × 10^8^ CFU mL^−1^ with a low LOD of 30 CFU mL^−1^. High specificity was confirmed by minimal interference from non-target bacteria (i.e., *E. coli*, *St. aureus*, *P. aeruginosa*, *S. typhimurium*, *Listeria monocytogenes*, and *Streptococcus thermophilus*), while the inherent photostability and low background of UCNPs supported reliable signal output in complex matrices. *Shigella* was directly detected in contaminated chicken samples without enrichment within 60 min, demonstrating suitability for rapid food safety analysis.

A hybrid nanostructure composed of biofunctionalized MNPs@UCNPs integrates magnetic enrichment with fluorescence-based signal readout for *Shigella* detection, improving sensitivity and reducing matrix interference [46]. In this system, enzymatic oxidation of 3,3′,5,5′-tetramethylbenzidine (TMB) to its oxidized form (oxTMB) induced fluorescence quenching at 545 nm via spectral overlap with UCNP emission. The biosensor could detect *Shigella* within 60 min with a low LOD of 32 CFU mL^−1^. High selectivity was confirmed by negligible responses to non-target bacteria, including *S. typhimurium*, *L. monocytogenes*, *St. aureus*, *P. aeruginosa*, *E. coli*, and *Str. thermophilus*. Validation in spiked chicken samples yielded recoveries of 97.8–108.5%, consistent with plate-counting results and an effective matrix interference suppression via magnetic separation. Beyond these fluorescence-based sensing platforms, more elaborate sandwich-type aptamer architectures have been developed to enable sequential target recognition and fluorescence signal readout. As illustrated in Figure 5b, the aptamer-based fluorescent biosensor for *S. sonnei* involves surface functionalization with an amine-modified capture aptamer (SS-4), binding of the target microbe, and subsequent fluorescence signal readout through hybridization with a Cy5-labeled detecting aptamer (SS-3).

SERS-based NP biosensors have been reported as optical tools for *Shigella* detection by exploiting NP-induced enhancement of characteristic molecular vibration signals. A SERS aptasensor based on AuNP dimers functionalized with a dual-functional europium (Eu) complex Raman reporter (4-mercaptobenzoic acid, 4-MBA) detects *S. sonnei* through NP-induced Raman signal enhancement [43]. The aptamer-functionalized nanostructure enabled highly specific bacterial capture and signal amplification, producing a strong SERS response at 1593 cm^−1^. The sensor exhibited a wide linear detection range from 10^1^ to 10^6^ CFU mL^−1^ and a low LOD of 10 CFU mL^−1^, outperforming PCR and fluorescence-based assays. High specificity was confirmed against other *Shigella* species (i.e., *S. dysenteriae*, *S. flexneri*, and *S. boydii*) and common FBPs (i.e., *S. typhimurium*, *St. aureus*, and *E. coli*), even at ten-fold higher concentrations. The aptasensor also demonstrated good repeatability, with a relative standard deviation (RSD) of 5.15% at 10^4^ CFU mL^−1^, and showed strong practical applicability in spiked food samples, resulting in recoveries of 92.6–103.8% consistent with plate-counting methods. AgNP-coated filter paper-based SERS substrates were also applied to detect *Shigella* [69]. Detection was achieved at concentrations as low as 10^1^ CFU, supported by the strong Raman enhancement factor of 4.63 × 10^6^ associated with the AgNP substrates. In aqueous and laboratory-simulated samples representative of food and environmental testing, the system showed good signal repeatability and chemical stability, allowing repeated acquisition of characteristic Raman fingerprints. In addition to sensing performance, the AgNP suspension displayed intrinsic antibacterial activity against *Shigella*, achieving bactericidal effects within 90 min at a minimum inhibitory concentration of 1300 μg mL^−1^, indicating the multifunctional potential of the developed SERS-active plasmonic substrates.

#### 4.1.3. Plasmonic Optical Biosensors

**Figure 6:**
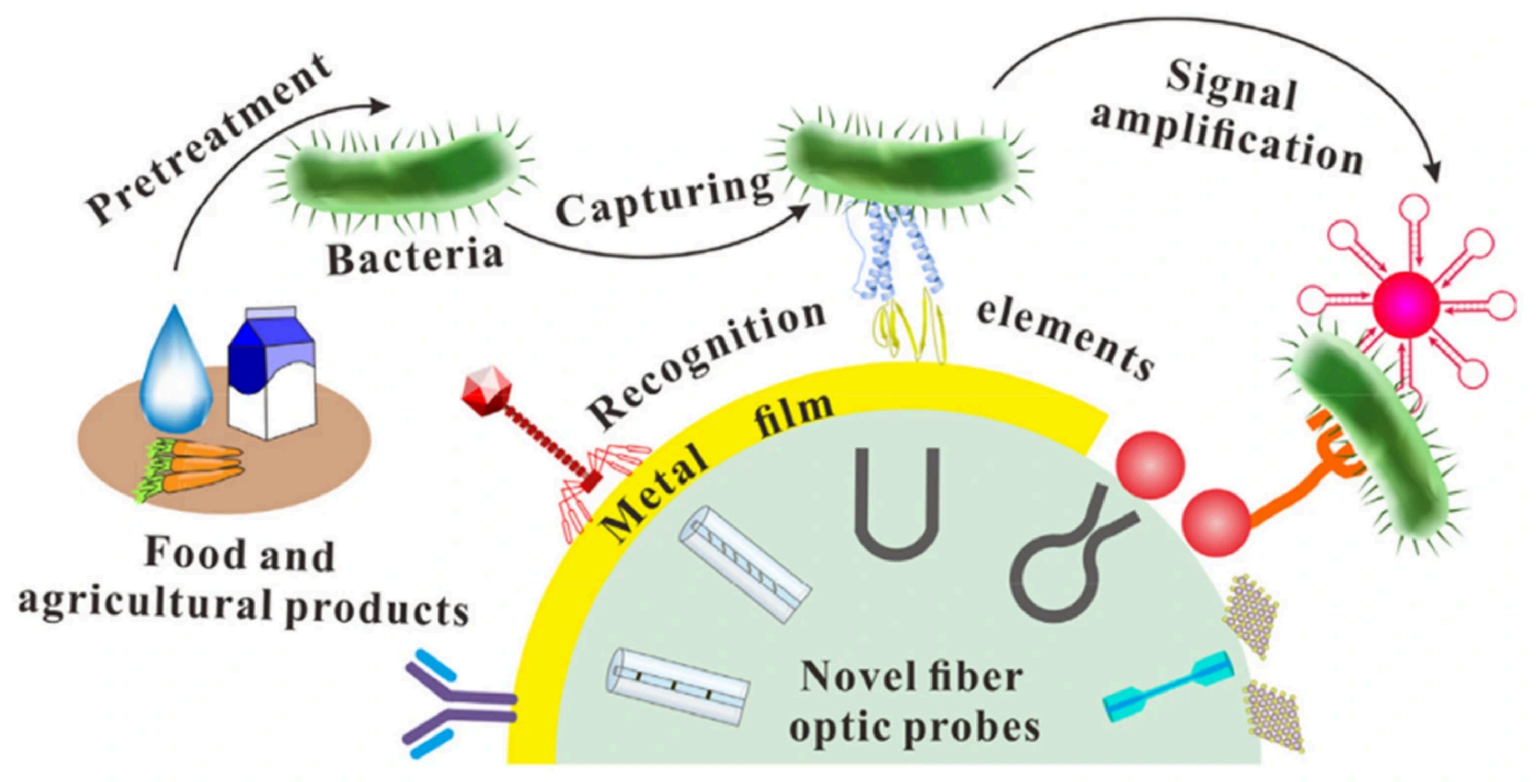
Schematic representation of the workflow of a fiber-optic-based biosensor for FBP detection. Reprinted from Gu et al. [27] with permission from the American Chemical Society.

As schematically illustrated in Figure 6, fiber-optic-based biosensors typically involve sample pretreatment, immobilization of biorecognition elements on metal-coated or nanostructured fiber probes, and signal transduction through SPR/LSPR-induced optical changes, which can be enhanced by NP-assisted surface modification and amplification strategies. LSPR-based optical fiber platforms have been reported for *Shigella* detection by integrating plasmonic nanomaterials with fiber-optic sensing. Multicore and tapered optical fiber probes functionalized with plasmonic nanomaterials have been developed for the selective detection of *Shigella* spp., demonstrating high sensitivity in liquid samples representative of food and water testing environments. In particular, Kumar et al. reported MoS_2_-functionalized multicore optical fiber probes exploiting LSPR for *Shigella* detection. The AuNP/MoS_2_-coated, surface-etched multicore fiber sensor achieved a broad linear range of 1–10^9^ CFU mL^−1^, a rapid 5 min response time, and an ultra-low LOD of 1.56 CFU mL^−1^, enabling reliable detection within the food-safety-relevant concentration range [67].

More recent studies have employed hybrid nanomaterial architectures to further promote LSPR-based optical fiber biosensors. Zhang et al. developed a multimodal LSPR-enhanced “crayfish-type” optical fiber sensor for ultra-sensitive detection of *S. sonnei*, integrating AuNPs with tungsten disulfide (WS_2_) thin layers and zinc oxide nanowires (ZnO-NWs) to amplify evanescent field interactions and increase bioreceptor loading. The label-free platform revealed a linear detection range of 1–1 × 10^7^ CFU mL^−1^, a sensitivity of 0.378 nm log^−1^ (CFU mL^−1^), and a low LOD of 4.78 CFU mL^−1^. The sensor demonstrated good reproducibility, stability, selectivity, and reusability and was successfully validated in food samples, including apples, cabbage, fish, and milk, signifying its applicability for food safety analysis [48]. Similarly, Gu et al. reported a three-stage S-tapered (3S) biophotonic optical fiber biosensor based on LSPR for rapid *Shigella* detection. The WaveFlex sensor, constructed using a core-mismatched multimode and four-core fiber architecture and functionalized with cerium oxide nanorods (CeO_2_-NRs), ZnO-NWs, and copper selenide@gold nanocomposites (Cu_3_Se_2_@Au-NCs), led to enhanced plasmonic coupling and bioreceptor immobilization. When functionalized with *S. sonnei*-specific antibodies, the platform demonstrated a high sensitivity of 0.28 nm per CFU mL^−1^, an ultra-low LOD of 0.96 CFU mL^−1^, and a rapid response time of 10 min, supporting its suitability for real-time food safety and environmental monitoring applications [81].

### 4.2. Electrochemical Nanoparticle-Based Biosensors

Electrochemical NP-based biosensors for *Shigella* detection are relatively limited in number but demonstrate high analytical sensitivity and applicability to complex matrices when appropriately validated. Reported platforms primarily rely on NP-modified electrodes to support selective biorecognition and quantifiable electrical signal changes following target binding.

An impedimetric aptasensor based on an AuNP-modified glassy carbon electrode detects *S. dysenteriae* by monitoring variations in charge-transfer resistance at the electrode interface following target binding [35]. The sensor showed a linear dynamic range from 10^1^ to 10^6^ CFU mL^−1^ with an LOD of 100 CFU mL^−1^. Dead *S. dysenteriae* cells produced no discernible signal, whereas high selectivity was observed toward non-target bacteria, including *St. aureus*, *E. coli* O157:H7, *S. enteritidis*, *S. flexneri*, and *Klebsiella pneumoniae*. The assay showed satisfactory analytical behavior without significant matrix interference when evaluated in water samples as well as pasteurized and unpasteurized skim milk. Target binding required 30 min, and results were consistent with real-time PCR validation. In the other study [70], a disposable electrochemical immunosensor employing an MWCNT/SA nanocomposite-modified screen-printed electrode was reported for *S. flexneri* detection. Under optimized conditions, the sensor displayed a linear response over 10^4^–10^11^ CFU mL^−1^ with an LOD of 3.1 × 10^3^ CFU mL^−1^. The incubation time required for immunocomplex formation was 40 min. High specificity was confirmed against *E. coli* O157:H7, *V. parahaemolyticus*, *S. pullorum*, *E. coli*, and *St. aureus*. Reproducibility testing yielded an RSD of 7.8%, and storage stability evaluation showed 95.16% signal retention after 2 weeks at 4 °C. Accuracy was verified through comparison with ELISA results.

A paper-based electrochemical aptasensor incorporating Ag-decorated nanobeads was developed for flexible and field-deployable detection of *S. dysenteriae* in food samples, based on target-induced modulation of interfacial electron-transfer behavior [38]. The sensing mechanism relies on target-induced modulation of interfacial electron-transfer behavior, which is monitored by electrochemical techniques, while integration with a near field communication (NFC)-enabled smartphone enables portable and on-site analytical readout. The platform enabled dual-mode operation using differential pulse voltammetry (DPV) and electrochemical impedance spectroscopy (EIS). For DPV, a linear range of 10^2^–10^8^ CFU mL^−1^ was achieved with an LOD of 90 CFU mL^−1^, while EIS measurements provided a wider linear range of 10^1^–10^9^ CFU mL^−1^ and a lower LOD of 8.09 CFU mL^−1^. The total analysis time was approximately 40 min. The sensor showed long-term stability of up to 28 days at 4 °C and facilitated direct detection in food samples without pre-enrichment. Furthermore, the NFC-enabled smartphone interface supports real-time data acquisition and extends the applicability of the electrochemical aptasensor for decentralized food safety screening. Electrochemical genosensing approaches have also been reported for *S. dysenteriae* using NP-based electrode modifications, providing direct nucleic acid detection through hybridization without the need for amplification. A PCR-free electrochemical genosensor based on a porous biochar/ultrathin MoS_2_ nanosheet composite with AuNPs detects *Shigella* DNA through direct hybridization, allowing sensitive detection in complex biological matrices without amplification steps [71]. The platform proved an LOD of 9.14 fM and an LOQ of 0.018 pM, with a linear response range from 0.01 to 100 pM (R^2^ = 0.999). The sensor distinguished target sequences from one-, two-, and three-base mismatched sequences and from non-target bacterial DNA, including *Haemophilus influenzae* and *S. typhimurium*, demonstrating high sequence specificity [71]. Validation in human plasma samples yielded recoveries of 98.0–101.3% with RSD values of 2.9–4.2%. Stability testing confirmed reproducible electrochemical responses over 15 days, supporting reliable performance in complex biological matrices.

Although nanomaterials can profoundly improve biosensor performance, no single material class is optimal for every analytical setting [2]. AuNPs simplify surface functionalization and optical signal generation, whereas carbon-based nanomaterials strengthen electron transfer and electrical sensitivity. MNPs are highly valuable for target enrichment and matrix cleanup before signal transduction, while quantum dots and related fluorescent nanomaterials facilitate highly sensitive detection and multiplex analysis [82]. These benefits coexist with practical limitations, including NP aggregation, variability in material properties, additional processing steps, and concerns regarding biocompatibility and large-scale implementation [83]. Hence, nanomaterial selection needs to account for the analytical objective, sample matrix, detection technique, and intended application, without considering LOD as the sole criterion [2,83].

## 5. Portable and Point-of-Care Nanoparticle-Based Biosensors for *Shigella*

Portable and point-of-care (POC) NP-based biosensing systems for *Shigella* detection have mainly relied on lateral flow (LF) and immunochromatographic formats that produce a rapid, visual signal without complex instrumentation. Colloidal gold immunoassay strips have been developed for simultaneous detection of *S. boydii* in food matrices such as bread, milk, and jelly, with visual LODs of approximately 10^6^ CFU mL^−1^ without enrichment and assay times of 5–10 min. LODs as low as 4 CFU mL^−1^ in food samples were recorded after short pre-incubation steps. These strip-based methods showed high specificity, with no detectable cross-reactivity toward non-target bacteria. The analytical results were comparable to those of traditional immunoassays, indicating effective application in complex food matrices [84].

AuNP-based lateral flow assays (LFAs) have also been reported for multiplex detection of *Shigella* spp. together with other FBPs, achieving successful identification in spiked milk samples and rapid visual results within a few minutes [32]. Multiple cross-displacement amplification (MCDA) has been combined with AuNP LF biosensors (LFBs) for nucleic-acid-level detection of *Shigella* spp., leading to improved sensitivity while retaining portability [85,86]. These MCDA-LFB systems reported analytical sensitivities down to 10 fg of genomic DNA per reaction or a few CFU per tube, with visual signal generation within 2–5 min after amplification [85]. Completion of the full assay required about 60 min [85]. Their suitability for POC and on-site testing was demonstrated by repeated observation of high specificity toward *Shigella*, with no interference from non-*Shigella* [85,86].

To improve detection capability while preserving suitability for field use, more advanced portable systems have incorporated multifunctional nanocomposites and alternative biorecognition elements. For whole-cell recognition, bacteriophage-assisted LFAs have become a viable substitute for antibody-based designs. Using NP-assisted catalytic signal amplification, a phage-based dual-signal LF biosensor showed high specificity and practical suitability for on-site food monitoring, making possible rapid visual identification of *Shigella* in aquatic food products [87]. CuS@Au nanocomposite-enhanced dual-mode immunochromatographic biosensors made detection possible for *S. sonnei* through both colorimetric and photothermal responses, providing a wide linear detection range and a 15 min test completion time. High specificity and stable signal behavior were observed under food-relevant testing conditions, and the dual-signal structure improved result dependability while retaining operational simplicity required for POC applications [88]. LF and immunochromatographic platforms represent the main portable NP-based approaches for *Shigella* detection in food safety applications. Regardless of the sensing format, selection of the biological target is an important consideration in NP-based biosensing.

## 6. Nanoparticle-Based Biosensors for *Shigella* Virulence Markers

Target choice directly influences how NP-based biosensors behave during *Shigella* detection. Whole-cell recognition and virulence-associated genes, especially *ipaH*, constitute the main targets for NP-based biosensors developed for *Shigella* detection [89,90,91]. PCR-free formats and isothermal amplification methods have also been described as alternatives intended to reduce procedural complexity for use outside conventional laboratory settings. Among reported targets, *ipaH* is the most commonly selected owing to its diagnostic relevance and relevance to genus-level *Shigella* identification in food-related samples [21,32,85,86].

The *ipaH* gene occurs in all *Shigella* species and is associated with genus-level identification with limited cross-reactivity toward non-*Shigella* enteric bacteria. NP-assisted biosensors directed toward *ipaH* have shown low LOD values at both DNA and cell-equivalent levels in biological and food samples [85,86]. Molecular sequence analyses have verified the diagnostic relevance of *ipaH*, accounting for its repeated selection in NP-based *Shigella* biosensing investigations [21,32]. The *spa* gene has also been described as an alternative virulence-related nucleic acid marker. Reports involving *spa* are restricted in number and largely confined to AuNP-based genosensing investigations [63].

A comparison between gene-level and whole-cell targeting indicates inherent differences in detection logic. Whole-cell targeting relies on direct recognition of intact bacterial cells, including *S. sonnei* [43] and *Shigella* spp. [34], without nucleic acid extraction, thereby shortening sample preparation and assay workflow [34]. These biosensors operate directly on intact cells without nucleic acid processing. Signal response may vary with matrix composition, physiological condition, or surface heterogeneity. Virulence markers like *ipaH* are the subject of gene-level sensing [85,86], *spa* [63], and *S. dysenteriae* DNA using PCR-free electrochemical genosensing [71]. These methods retain specificity despite differences in bacterial viability. Gene-level detection emphasizes confirmatory identification. Whole-cell detection emphasizes operational simplicity and short assay timelines.

Several NP-based *Shigella* biosensors exclude amplification steps altogether. Unmodified AuNP optical genosensors targeting *spa* have been described for direct hybridization-based detection without amplification [63]. A biochar/MoS_2_/AuNP electrochemical genosensor has also been described for PCR-free detection of *S. dysenteriae* DNA in complex samples [71]. Enzyme-free signal generation using DNAzyme-based colorimetric sensing has been described for *S. flexneri* [39]. Direct whole-cell detection of *S. dysenteriae* has also been achieved using AuNP-modified impedimetric aptasensors operating without nucleic acid amplification, based on target-induced changes in interfacial electron-transfer resistance [35]. These PCR-free formats reduce procedural steps and shorten analysis time, consistent with food-related *Shigella* biosensing requirements. The analytical value of a biological target cannot be assessed independently of the sensing strategy used for its detection.

## 7. Multiplex and Multimodal Nanoparticle-Based Biosensing for *Shigella*

Food analysis sometimes requires detection of *Shigella* in samples that also contain other foodborne bacterial pathogens or confirmation based on more than one signal output. For this reason, multiplex and multimodal NP-based biosensors have been described for food-related applications. These biosensors emphasize simultaneous detection and independent signal readout in complex food samples, without seeking additional reductions in single-analyte LOD values [2,92,93,94].

AuNP-assisted LFBs were applied to multiplex nucleic acid detection. In combination with multiple cross-displacement amplification, these assays generated visual signals for *Shigella* spp. and additional bacterial targets through spatially separated test lines, with specificity maintained during analysis [85,86]. AuNP-based LFAs were likewise applied to the detection of *Shigella* and *Salmonella* genera in contaminated food samples. In these assays, a multiplex visual signal was obtained without compromising assay mobility or procedural simplicity [32]. Multiplex detection at the protein and whole-cell levels has been accomplished using immunochromatographic and microarray-based techniques. *S. flexneri* was detected in food samples using fluorescent microsphere-labeled immunochromatographic strips, while signal differentiation was noted during testing [95]. Several FBPs, including *Shigella*, have been detected using mesoporous silica NP-assisted microarray techniques based on fluorescence signals acquired from arrayed targets. The setup combined a functionalized biochip with mesoporous silica NP-streptavidin-antibody assemblies for pathogen capture and fluorescence signal in several targets [29]. Multimodal sensing has been investigated by using multiple signal outputs in a single experiment. Dual-signal sandwich biosensors using truncated aptamers produced complementary optical signals for *S. sonnei*, reducing ambiguity during interpretation in complex samples [37]. UCNP-based sensor arrays have been used for discrimination of multiple pathogenic bacteria, including *Shigella*, based on pattern-dependent fluorescence responses instead of single-target quantification [45,96].

## 8. Nanoparticle-Based Biosensors for *Shigella* in Real Food Matrices

Table 1 summarizes NP-based biosensors reported for detection of *Shigella* spp. in real food matrices, including target species, food categories, sensing formats, nanomaterials, operating principles, and reported detection metrics. Validation using real food samples has covered a wide range of commodities, including meat products (e.g., chicken, beef, and pork), seafood (e.g., fish, shrimp, squid, clam, and smoked salmon), dairy products (e.g., milk and skim milk), fresh produce (e.g., lettuce, cabbage, apple, and kelp), and processed foods (e.g., bread, jelly, canned tuna, and chocolate). By and large, these studies demonstrate that NP-based biosensors can maintain high sensitivity, selectivity, and robustness despite the intrinsic complexity and matrix interferences associated with real foods. From a target perspective, generic *Shigella* spp. sensors are mostly applied to screening scenarios in chicken, milk, beef, and seafood. These systems, often fluorescence-based or LFA-type platforms, typically achieve LODs in the range of ~10–30 CFU mL^−1^ with assay times ≤ 60 min, making them suitable for rapid on-site monitoring. In contrast, species-specific sensors (i.e., *S. flexneri*, *S. sonnei*, *S. dysenteriae*, and *S. boydii*) generally demonstrate lower LODs and wider linear ranges, reflecting the higher affinity and specificity of aptamer- or antibody-based recognition elements.

Among the species, *S. flexneri* and *S. sonnei* are the most extensively evaluated in real foods. Detection of *S. flexneri* in milk, fish, and lettuce reaches single-digit CFU mL^−1^ using LSPR and SERS/fluorescence configurations, with lower detection thresholds than colorimetric assays. For *S. sonnei*, detection in canned tuna, chicken, milk, fruits, and vegetables falls within 4–10 CFU mL^−1^ using LSPR and SERS configurations. In comparison, *S. dysenteriae* sensors, often electrochemical, display slightly higher LODs (8–100 CFU mL^−1^) but remain highly effective in water, milk, eggs, and meat products. LF immunoassays show acceptable sensitivity when enrichment steps are applied, despite the limited number of reports addressing detection of *S. boydii*. These assays are therefore mainly associated with qualitative or semi-quantitative screening (Table 1).

Sensor modalities used for *Shigella* detection in food matrices differ in analytical behavior. Optical sensors based on fluorescence, upconversion fluorescence, LSPR, and SERS typically exhibit broader linear ranges and lower LODs. These properties are linked to signal amplification effects that come from plasmonic nanostructures, AuNPs, UCNPs, and nanozymes. In the reported studies, optical biosensing platforms based on fluorescence, upconversion fluorescence, LSPR, and SERS generally showed lower susceptibility to matrix interference from proteins, lipids, and other coexisting constituents in food matrices such as milk, shellfish, and ready-to-eat foods (Table 1). Electrochemical sensors provide a balance between analytical sensitivity and convenience of use. They are primarily based on EIS or DPV and are used on paper-based substrates or AuNP-modified electrodes. These designs are characterized by mobility, resistance to matrix effects, and assay times (30–40 min). LOD values are often greater than those derived from optical outputs. LFAs prioritize simplicity and speed, providing visual readouts in 5–10 min. This fact is highly accompanied by decreased sensitivity at extremely low target concentrations. Significant increases in LFA sensitivity have resulted from the use of nanozymes, magnetic separation procedures, and signal-amplifying nanomaterials. Detection levels of seafood and processed food are comparable to those found using more measurement-intensive techniques [29,31,34,35,37,38,39,43,45,46,48,49,65,66,84,87,96,97,98,99].

The choice of nanomaterial has a significant impact on the results obtained from actual food samples. MNP-based biosensing platforms are advantageous for protein- or lipid-rich food matrices (e.g., dairy and meat products) because magnetic separation and preconcentration can reduce matrix interference before signal detection. Near-infrared (NIR) excitation is made possible by UCNPs, which lessens interference from food autofluorescence. Plasmonic nanomaterials (e.g., AuNPs), Ag-based structures, and gold-copper bimetallic NPs (AuCu) nanoclusters improve optical responsiveness through LSPR or SERS effects. Linear ranges of *Shigella* for these biosensors were from 10^2^ to 10^7^/10^8^ CFU mL^−1^. The most efficient sensing systems of *Shigella* in milk, fish, and lettuce, including SERS-, LSPR-, and DNA walker-based dual-mode types, showed LODs as low as 1–5 CFU mL^−1^. Most assays are completed within 60 min. LFAs and basic colorimetric sensors generate results within 20–40 min, whereas more complex multimodal or DNA-based systems may require up to 180 min while providing greater sensitivity. Many systems report recovery values of about 90–110% in different matrices, indicating practical use of NP-based biosensors in real food samples. Specificity assessments also show very low cross-reactivity with non-target FBPs, even at elevated concentrations. This observation applies strongly to aptamer- and antibody-based systems, which can differentiate target *Shigella* species from other pathogens in multi-pathogen environments representative of real food samples (Table 1).

Comparative analysis shows that no single biosensor format fits all food-testing scenarios. Optical platforms achieve the lowest LODs and support confirmatory analysis, especially in complex matrices with strong signal amplification effects. Electrochemical systems provide a balance between sensitivity and operational simplicity, making them appropriate for routine and portable testing. LFAs, despite their lower analytical sensitivity, offer rapid preliminary screening under field-based, resource-limited conditions. Therefore, selection of biosensor platforms should consider not only analytical performance metrics such as LOD but also application requirements, including assay time, portability, and matrix complexity.

**Table 1:** Analytical performance of NP-based biosensors for *Shigella* detection evaluated in real food samples.

*Shigella* spp. (Target)	Real Food Matrix	Sensor Type ^1^	NPs/Nanomaterial ^2^	Operational Conditions ^3^	Detection Mechanism ^4^	Linear Range (CFU mL^−1^)	LOD (CFU mL^−1^)	Assay Time (min)	Recovery (%)	Ref.
*Shigella* spp. (whole-cell)	Chicken	Fluorescence	UCNPs + AuNPs	980 nm excitation; Aptamer-mediated whole-cell recognition; No enrichment	FRET-based fluorescence recovery	1.2 × 10^2^–1.2 × 10^8^	30	≤60	-	[34]
*Shigella* spp. (whole-cell)	Squid meat, Kelp, Bullfrog meat, Clam meat	LFA (phage-based, dual-pathogen)	Mannose-modified Fe_3_O_4_ nanozymes (Man-sFe_3_O_4_ NPs)	Magnetic separation using Man-sFe_3_O_4_ NPs; Acidic nitrocellulose membrane modification (pH 3); Catalytic color development with DEPDA (naphthol + diamine + H_2_O_2_)	Phage-mediated capture + nanozyme-catalyzed colorimetric LFA readout	10^1^–10^5^	14.1	≤60	96.5–107.7	[87]
*Shigella* spp. (whole-cell)	Milk, Ground beef	Fluorescence sensor array	Surface-chemistry-modified UCNPs	Differential surface–bacteria interactions; No enrichment; No nucleic acid amplification	Pattern-recognition-based fluorescence response (sensor array discrimination)	-	-	Rapid (exact time not specified)	-	[45]
*Shigella* spp. (whole-cell)	Chicken	Fluorescent aptasensor	Fe_3_O_4_ MNPs@UCNPs + HRP	Magnetic separation; TMB; 37 °C	Aptamer–cell binding → HRP/TMB inner filter effect → UCNP fluorescence change	2.3 × 10^2^–2.3 × 10^7^	32	~60	97.8–108.5	[46]
*S. dysenteriae* (whole cell, live)	Water, Skim milk (Un/Pasteurized)	Impedimetric aptasensor (EIS)	AuNP-modified GCE	pH 7.4; Room temperature (RT); 30 min binding	Aptamer–cell binding → ↑ charge transfer resistance (Rct)	10^1^–10^6^	100	~30	93.3–132.9	[35]
*S. dysenteriae* (whole-cell)	Eggs, Chicken, Pork, Shrimp	Electrochemical (paper-based aptasensor)	Silver (Ag)-decorated nanobeads	Aptamer-based whole-cell recognition; Dual electrochemical readout (DPV and EIS); Portable, Flexible format	Electrochemical signal change upon target–aptamer binding	10^2^–10^8^ (DPV); 10–10^9^ (EIS)	90 (DPV); 8.09 (EIS)	~40	-	[38]
*S. dysenteriae* (gene-level, *ipaH*)	Milk	AuNP-amplified microcantilever array biosensor	AuNPs	ssDNA probe immobilization on microcantilever; AuNP-assisted cascade hybridization; Piezoresistive deflection readout; No PCR or culture; Flow rate 0.40 μL s^−1^; RT	Nanomechanical deflection induced by DNA hybridization amplified by AuNP mass loading	24–1.2 × 10^5^	9	<60	Not reported; Good agreement with ddPCR	[98]
*S. flexneri* (whole-cell, multiplex context)	Tap water, Milk, Beef	Fluorescence (upconversion-based, nonspecific multiplex sensing)	Guanidine-functionalized UCNPs	980 nm NIR excitation; Direct incubation of bacteria with guanidine-functionalized UCNPs; No bioreceptor immobilization; No enrichment step	Bacteria-induced upconversion fluorescence enhancement	10^3^–10^8^	2.5 × 10^2^	~30	77.8–105.6% (Tap water), 71.4–111.4% (Milk), 73.4–110.9 (Beef)	[96]
*S. flexneri* (whole-cell)	Fish, Shrimp, Chicken	Colorimetric DNAzyme aptasensor	SWCNT (support); hemin–G4 DNAzyme	RT; ABTS/H_2_O_2_; Read at 420 nm	Target-induced DNAzyme activity (color change)	10^3^–10^7^	51	~30–40	-	[39]
*S. flexneri* (whole-cell)	Milk, Fish, Fruit juice, Chocolate	Biophotonic fiber-optic biosensor (LSPR-based)	MoS_2_ nanosheets + ZnO nanowires + AuCu nanoclusters	Three-S-tapered optical fiber; Antibody-functionalized surface; LSPR wavelength shift measurement; Optimal pH ≈ 7.4; 37 °C; No enrichment	LSPR-based refractive index change induced by antigen–antibody binding	1–10^8^	4.412	≤60	~99	[99]
*S. flexneri* (whole-cell)	Smoked salmon (ready-to-eat seafood)	Colorimetric aptasensor	AuNPs functionalized with DNA aptamer	Aptamer-modified AuNPs; NaCl-induced aggregation; Incubation at 37 °C; No enrichment or DNA extraction; Visual readout and UV–Vis (A_700_/A_520_)	Target-induced AuNP aggregation causing color change (red → purple/blue)	10^2^–10^6^	80	≤20	88.51–110.20	[31]
*S. flexneri* (whole-cell)	Water, Milk, Lettuce	DNA walker-driven dual-mode aptasensor (SERS + fluorescence (FL))	Fe@SiO_2_@QDs (fluorescent probe); AgR6G@SiO_2_ (SERS tag)	Zn^2+^-activated DNAzyme; 37 °C incubation; Magnetic separation	Aptamer binding triggers DNA walker → H1 cleavage → fluorescence recovery + SERS signal	1.3 × 10^1^–2 × 10^8^ (FL); 8–8 × 10^8^ (SERS)	11 (FL); 3 (SERS)	~180	97.6–107.3	[66]
*S. flexneri* (whole-cell)	Milk	Fluorescent aptasensor	Graphene oxide (GO)	37 °C; ApaI-assisted recycling	Aptamer binding + GO quenching + enzymatic signal amplification	5 × 10^2^–1 × 10^9^	100	~60	-	[65]
*S. sonnei* (whole cell, heat-killed)	Canned tuna	Fluorescent antibody–aptamer microarray (7FP-biochip)	Mesoporous silica NPs–fluorescein–streptavidin–antibodies (MSNs-Flu-SA-Abs)	PBS pH 7.4; RT; 60 min incubation	Sandwich immunoassay, Fluorescence amplification	10–10^5^	6 (Buffer); 25 (Food)	~120	85.7–134.8	[29]
*S. sonnei* (whole-cell)	Water, Milk, Lettuce	Dual-signal sandwich aptasensor (colorimetric (CM) + SERS)	Fe_3_O_4_@SiO_2_@Ag (capture); Cu-TCPP(Fe)/AuNPs (signal)	37 °C incubation; Magnetic separation	Aptamer–cell–aptamer sandwich; TMB oxidation + SERS	10^2^–10^7^ (CM); 10^1^–10^8^ (SERS)	37 (CM); 7 (SERS)	~95	88.98–112.92 (CM); 98.40–103.95 (SERS)	[37]
*S. sonnei* (whole-cell)	Apple, Cabbage, Fish, Milk	LSPR optical fiber immunosensor	AuNPs/WS_2_/ZnO NWs	λ-shift readout; pH 7.4, 37 °C tested	Antibody–cell binding → RI change (LSPR shift)	1–10^7^	4.78	-	88.1–113.3	[48]
*S. sonnei* (whole-cell)	Chicken breast, Milk	SERS	AuNP dimers + Eu-complex (4-MBA reporter)	Aptamer-mediated bacterial capture; Raman excitation at 785 nm; No enrichment	SERS signal enhancement	10^1^–10^6^	10	-	92.6–103.8	[43]
*S. sonnei* (gene-level, DNA)	Milk, Potato, Chicken, Lobster, Fish, Egg	Multimodal electrochemical–ICP-MS biosensor	AuNPs	DNA extraction from food matrices; AuNP-assisted signal amplification; Electrochemical readout coupled with ICP-MS quantification; No culture enrichment	NP-assisted electrochemical detection combined with ICP-MS elemental signal amplification	10–10^10^ copy mL^−1^ (DNA (gene) level)	1 copy mL^−1^	-	93.2–107.6	[49]
*S. boydii* (whole cell)	Bread, Milk, Jelly	Lateral flow immunochromatographic strip (ICS)	Colloidal AuNPs–McAbs	Visual readout; RT; Pre-enrichment optional	Sandwich immunoassay (AuNP–Antibody (Ab)–cell–Ab)	-	10^6^ (direct); 4 after enrichment	5–10	-	[84]

^1^ LSPR: Localized surface plasmon resonance; SERS: Surface-enhanced Raman spectroscopy; ICP-MS: Inductively coupled plasma–mass spectrometry; LFA: Lateral flow assay. ^2^ AuNPs: Gold nanoparticles, UCNPs: Upconversion nanoparticles; Man-sFe_3_O_4_ NPs = Mannose-functionalized superparamagnetic Fe_3_O_4_ nanoparticles; MNPs: Magnetic nanoparticles; HRP: Horseradish peroxidase; GCE: Glassy carbon electrode; SWCNT: Single-walled carbon nanotube; hemin–G4: Hemin–G-quadruplex DNAzyme; MoS_2_: Molybdenum disulfide; Fe@SiO_2_@QDs: Iron oxide core–silica shell nanoparticles decorated with quantum dots; AgR6G@SiO_2_: Silver nanoparticles loaded with rhodamine 6G and coated with a silica shell; WS_2_/ZnO NWs: Tungsten disulfide combined with zinc oxide nanowires; Eu-complex (4-MBA reporter): Europium complex labeled with 4-mercaptobenzoic acid as the SERS reporter; TCPP(Fe)/AuNPs: Iron–tetra(4-carboxyphenyl)porphyrin immobilized on gold nanoparticles; McAbs: Monoclonal antibodies. ^3^ DEPDA: N,N′-diethyl-*p*-phenylenediamine; TMB: 3,3′,5,5′-Tetramethylbenzidine; NIR: Near-infrared; DPV: Differential pulse voltammetry; EIS: Electrochemical impedance spectroscopy; ABTS: 2,2′-azino-bis(3-ethylbenzothiazoline-6-sulfonic acid), ApaI: Restriction endonuclease ApaI; PBS: Phosphate-buffered saline. ^4^ FRET: Förster resonance energy transfer; H1 cleavage: DNA walker-mediated cleavage of H1 strand; RI change: Refractive index change.

## 9. Challenges, Limitations, and Future Perspectives

Despite substantial progress in NP-based biosensors for *Shigella* detection, several challenges, including electrochemical and LF, continue to limit their broader implementation in routine food safety monitoring. A primary shortcoming is the gap between laboratory-scale sensor performance and real-world applicability. Although many platforms demonstrate excellent sensitivity under controlled conditions, their analytical performance can be influenced by complex food matrices containing fats, proteins, salts, and indigenous microbiota [100,101]. Recovery studies summarized in Table 1 generally report acceptable quantitative agreement (typically ~90–110%), but matrix-specific effects are still insufficiently standardized across sensor types and food categories, complicating direct comparison and regulatory translation.

Assay integration and daily operations are still problematic in developing *Shigella* biosensors. Implementation of optical sensing techniques, including dual-mode fluorescence detection, LSPR, and SERS, highly depends on specific nanomaterial modification, multi-stage production, or technical optical equipment. These specifications restrict scalability and make low-cost applications in well-equipped laboratory environments less feasible. On the other hand, although quick tools like LFAs and simple colorimetric assays are more portable and easier to use, they showed higher LODs for *Shigella* until combined with enrichment procedures, signal amplification, or nanozyme-assisted enhancement [44,102]. Therefore, there is a necessity to balance analytical sensitivity with ease of use in the design of *Shigella* biosensors.

Further restrictions are introduced by target choice. Even though whole-cell detection contributes to the direct identification of whole bacteria, results can be different depending on the presence of living but non-culturable cells, surface heterogeneity, and physiological conditions. Since DNA extraction or extra processing is often necessary to avoid limits on quick implementation apart from laboratory settings, gene-level detection targeting virulence indicators like *ipaH* produces excellent specificity and repeatable results. Even though PCR-free genosensing systems simplify the process, there are still a few reports to emphasize the necessity for more extensive validation using a variety of food matrices and contamination conditions [1,26,103,104].

Meaningful comparison is hampered by the significant diversity in linear ranges, LODs, assay length, and validation technique reported in investigations addressing identical sensor categories. Adopting common evaluation standards would facilitate regulatory consideration for usage with actual food samples and improve comparability between studies [105]. Combining multiplex and multimodal systems is a potential approach since it can increase the reliability of results while enabling parallel screening and confirmation detection of several FBPs in a single test. Future research should also focus on analytical performance, measurement uniformity, stability during prolonged storage and recurrent usage, and scale-up viability. Many nanomaterial-based sensors demonstrated strong short-term sensitivity, yet information on batch-to-batch reproducibility and long-term stability is often lacking.

Current limitations differ across sensing platforms and require targeted research strategies. Optical biosensors face challenges related to instrument complexity, dependence on precise nanomaterial engineering, and confined scalability for routine field deployment. Future work should focus on simplified optical configurations, development of stable and cost-effective nanomaterials, and integration of miniaturized detection systems for portable use. Electrochemical biosensors show compatibility with on-site applications but are affected by electrode fouling, signal instability in complex food matrices, and variability in surface modification. These issues require development of antifouling electrode materials, standardized fabrication protocols, and improved long-term stability under real sample conditions. LF systems are constrained by limited sensitivity at low bacterial concentrations, which restricts their role in confirmatory analysis. Research directions should therefore include signal amplification strategies, integration with nucleic acid amplification methods, and improved quantitative readout capability. Across all platforms, future studies should emphasize validation in diverse and realistic food matrices, establishment of standardized performance criteria, and translation of laboratory-scale designs into scalable and user-oriented systems for routine food safety monitoring.

Hybrid and hierarchical NPs offer a useful route for future *Shigella* biosensors. The assembly of two or more nanomaterials in an ordered multilevel structure can place target capture, signal amplification, and transduction in the same sensing system. Their highly accessible surface area, adjustable porosity, and efficient electronic or ionic charge transfer can increase probe loading, biomolecular contact, and signal intensity and may reduce matrix interference in complex food samples [106]. Even though direct studies on *Shigella* are scarce, these NPs could permit more sensitive, multiplex, and stable assays for routine food analysis. Future studies should investigate fabrication reproducibility, material stability, cost, and analytical performance in real food matrices before large-scale use.

The use of artificial intelligence (AI) and machine learning (ML) may improve the future performance of NP-based biosensors for *Shigella* detection, mainly for the analysis of complex sensing data. AI and ML operate on signals and datasets produced by analytical platforms and can improve automated interpretation, pattern recognition, classification, and prediction [61]. For NP-based *Shigella* biosensors, these methods may be applied to colorimetric images, electrochemical signals, Raman spectra, and multiplex datasets, although direct evidence from *Shigella*-specific AI-assisted systems is currently limited. Convolutional neural networks can process image-based readouts, while support vector machines and random forests can classify spectral or electrochemical data and extract informative features from noisy backgrounds [61,107]. In SERS-based FBP analysis, automated feature extraction, ML-based classification, dimension reduction, and multimodal data fusion have improved bacterial discrimination and analytical accuracy, indicating a relevant pathway for future *Shigella* sensing studies [108]. AI-assisted analysis may also reduce subjective visual interpretation and improve rapid analysis via smartphones and point-of-care devices. Coupling sensor data with cloud and Internet-of-Things (IoT) systems may enable contamination-trend analysis, real-time risk assessment, and large-scale food monitoring [107]. Yet, reliable application requires large and well-annotated datasets, consistent signal quality, standardized spectral libraries, and validation in food matrices, instruments, and laboratories [107,108]. Dataset shift, model bias, narrow interpretability, and regulatory assessment also require careful consideration. As a result, AI and ML should currently be viewed as complementary tools for signal analysis, classification, and risk assessment while established sample preparation, biosensing, and confirmatory procedures still constitute the analytical basis of *Shigella* detection.

Future development of NP-based biosensors for *Shigella* detection should place greater emphasis on scalable manufacturing, standardized fabrication protocols, and quality-controlled production to improve reproducibility and commercial translation [53,55]. Cost-effective sensing materials, simplified fabrication procedures, compatibility with high-throughput manufacturing, and user-friendly device formats may reduce production costs while preserving analytical performance in routine food monitoring [56,57,58]. Standardized validation using naturally contaminated food samples, discrimination between viable and dead cells, and improved cross-platform consistency should receive additional attention before large-scale implementation [61]. Recognition elements with low production cost and scalable synthesis, including molecularly imprinted polymers, peptides, and engineered affinity probes, may support practical deployment, although additional validation in complex food matrices is still required [54]. These advances may accelerate regulatory acceptance and routine application of NP-based biosensors for food safety monitoring [59].

The transition of NP-based *Shigella* biosensors from proof-of-concept investigations to routine food safety surveillance tools will depend on resolving these practical limitations, simplifying manufacturing processes, and improving ease of use in practical settings.

## 10. Conclusions

Recent developments in NP-based biosensors to detect *Shigella* spp. in food safety applications were introduced. Utilizing NPs led to an improvement in the sensitivity, selectivity, and signal transduction in optical, electrochemical, and hybrid biosensors used for *Shigella* contamination diagnosis in real food matrices. Electrochemical and LF systems demonstrated operational simplicity and portability suitable for on-site screening, whereas optical techniques such as fluorescence-, SERS-, and LSPR-based systems achieved the lowest LODs and performed well in complex food samples. Whole-cell recognition allowed rapid and straightforward detection without the need for extensive sample processing, whereas gene-focused methods directed at stable virulence markers, mainly *ipaH*, contributed to accurate identification at the genus level. The practical value of these biosensing devices was demonstrated through validation tests carried out in several food matrices, which showed acceptable recovery rates, minimal cross-reactivity, and close agreement with reference methods. NP-based biosensing therefore represents an effective and adaptable means for tracking *Shigella* in food systems and provides a defined pathway for moving laboratory-scale biosensing developments toward food safety surveillance applications.

## Data Availability

No new data were created or analyzed in this study. Data sharing is not applicable to this article.

## Abbreviations

The following abbreviations are used in this manuscript:

4-MBA	4-mercaptobenzoic acid
AgNPs	silver nanoparticles
AI	artificial intelligence
AQMS	anthraquinone-2-sulfonic acid monohydrate sodium salt
AuNPs	gold nanoparticles
CeO_2_-NRs	cerium oxide nanorods
CFU	colony-forming unit
Cu_3_Se_2_@Au-NCs	copper selenide@gold nanocomposites
CuS	copper sulfide
Cy5	cyanine 5
DNAzyme	deoxyribonucleic acid enzyme
DPV	differential pulse voltammetry
EIS	electrochemical impedance spectroscopy
ELISA	enzyme-linked immunosorbent assay
Eu	europium
FBP(s)	foodborne pathogen(s)
fM	femtomolar
FRET	Förster resonance energy transfer
GNPs	gold nanoparticles
ICP-MS	inductively coupled plasma-mass spectrometry
IoT	Internet of Things
ipaH	invasion plasmid antigen H
LF(A)	lateral flow (assay)
LF(B)	lateral flow (biosensor)
LOQ	limit of quantification
LOD(s)	limit(s) of detection
(L)SPR	(localized) surface plasmon resonance
MCDA	multiple cross displacement amplification
ML	machine learning
MNPs	magnetic nanoparticles
MWCNT/SA	multiwalled carbon nanotube-sodium alginate
NFC	near field communication
NIR	near-infrared
NPs	nanoparticles
OD600	optical density at 600 nm
(ox)TMB	(oxidized) 3,3′,5,5′-tetramethylbenzidine
(q)PCR	(quantitative) polymerase chain reaction
pM	picomolar
POC	point-of-care
RSD	relative standard deviation
SERS	surface-enhanced Raman spectroscopy
spa	*Shigella* plasmid antigen gene
UCNPs	upconversion nanoparticles
VBNC	viable-but-non-culturable
WS_2_	tungsten disulfide
ZnO-NWs	zinc oxide nanowires
